# Smiling in Pain: Explorations of Its Social Motives

**DOI:** 10.1155/2013/128093

**Published:** 2013-08-19

**Authors:** Miriam Kunz, Kenneth Prkachin, Stefan Lautenbacher

**Affiliations:** ^1^Department of Psychology, Physiological Psychology, Otto-Friedrich University Bamberg, Markusplatz 3, 96045 Bamberg, Germany; ^2^Department of Psychology, Health Psychology, University of Northern British Columbia, 3333 University Way Prince George, BC, Canada V2N 4Z9

## Abstract

Studies of facial responses during experimental and clinical pain have revealed a surprising phenomenon, namely, that a considerable number of individuals respond with a smile. So far, it is not known why smiling occurs during pain. It is possible that the “smile of pain” is socially motivated (e.g., reinforcing social bonds while undergoing an unpleasant experience). The present studies were conducted in an attempt to address the role of social motives in smiling during pain. In two studies, we varied the quantitative (level of sociality) and qualitative (properties of the relationship between interactants) components of the situations in which participants received painful stimulation. Participants' faces were video-recorded and the occurrence of smiling was assessed. The occurrence of smiling differed depending on stimulus intensity and the properties of the relationship between interactants. Smiling occurred more often during the painful compared to nonpainful stimulation. Whereas the presence of a stranger (experimenter) reduced the smiling behavior, the presence of an intimate other increased it. Slight variations in the level of sociality, however, had no effect on the degree of smiling. Social motives possibly aimed at strengthening social bonds and thus ensuring social support appear to underlie smiling during pain.

## 1. Introduction

There is now a substantial body of literature showing that people in pain evince a number of specific facial movements [1], with a set of at least three or four distinct facial actions showing a close linkage to pain [2, 3]. However, this core set of pain-indicative facial actions is often blended with a range of seemingly incongruent expressions. Among the most frequently reported—in experimental as well as clinical studies [2, 3]—is the action of the zygomatic major muscle, which pulls the lip corners upward obliquely [4]. This finding appears surprising because the action of the zygomatic major is better known as the principal movement in the most common human facial expression—the smile [5, 6]. Given that facial expressions occurring during pain have been shown to not only impact social interactions (by eliciting solicitous behaviors and empathic responses in observers) but also impact pain diagnostic and pain treatment we believe it is important to investigate this seemingly incongruent facial expression, namely, “smiling during pain.” Although one might initially associate a smile with happiness or at least with a marker of a positive, non-painful affective state, it has been shown that there are different types of smiles and, interestingly, many of them appear not to be signs of felt happiness [5, 7]. 

In a recent comprehensive article on smiling [8], the authors suggest that there are three different types of smiles which have discrete functions. One group of smiles, labeled “*enjoyment smiles*,” is believed to indeed be readouts of positive emotions like happiness. Another group, labeled as “*dominance smiles*,” refers to the smiles that reflect a dominant social status or control. The third group, labeled as “*affiliative smiles*,” refers to those smiles that express positive social motives and serve the purpose of creating and maintaining social bonds [8]. To which of these categories do smiles occurring during pain belong? By definition, pain is an unpleasant experience (IASP, 1986) and thus, it seems unlikely that smiles occurring during pain are *enjoyment smiles *that are readouts of a positive emotion. However, from the perspective of the opponent process theory [9]—which assumes that the arousal of a primary affect (e.g., pain) also initiates the arousal of a consequent opponent process (e.g., positive affect)—smiling during the experience of pain could indeed reflect the arousal of an opponent positive affective state, arising from an inherent homeostatic adjustment to the arousal of a negative affective state. Smiling during the experience of pain could indeed reflect the arousal of an opponent positive affective state arising from an inherent homeostatic adjustment to the arousal of a negative affective state. 

However, it seems even more likely that smiles occurring during pain belong to the group of *affiliate smiles* that express positive social motives. These social motives are believed to be the creation and maintenance of social bonds [8]. In the context of pain, which is often a threatening, distressful, and highly arousing experience [10], social bonding might be especially important in order to engage social support. In the present studies, we addressed the question of whether smiles occurring during the experience of pain really belong to this group of socially motivated *affiliate smiles*. To answer this question, we tested participants in different social settings. If smiling during pain does indeed serve social motives, then smiles in response to painful stimulation should be significantly affected by social context manipulations. 

The empirical definition of “social” in facial expression research, however, is rather vague and has varied from simply altering the presence of another person (e.g., alone versus with the experimenter) to manipulating specific aspects of the social interaction (e.g., eye contact) [11] These variations can be arranged on a continuum ranging from no sociality (being alone) to moderate (nonvisual interaction) and high sociality (visual interaction). Besides these more quantitative variations in the level of sociality, social situations can also differ qualitatively [11] depending on the type of relationship between interactants. In the present studies, we investigated the impact of varying levels of sociality as well as of different properties of the social relationship on the occurrence of smiling during pain. In study 1, which employed a between-subject design, we investigated whether even slight variations in the degree of sociality affect the occurrence of smiling during pain by comparing situations with high and moderate sociality. In both conditions, an experimenter was always present. In the high sociality condition the experimenter did and in the low sociality condition, the experimenter did not have visual contact with the participant. In study 2, which employed a within-subject design, we not only modified the degree of sociality but also varied properties of the social relationship. Participants experienced pain while alone, with a significant other, and with a stranger. 

In addition to applying painful stimulation, we also applied nonpainful stimuli in order to assess the degree to which smiling was indeed specific to painful experiences.

## 2. Methods

Two studies were conducted (see Table 1). In study 1, involving a slight variation in the level of sociality, an experimenter always remained in the room either facing the participant (visual interaction = high sociality) or seated behind a computer screen that blocked the experimenter's face (nonvisual interaction = moderate sociality). In study 2, the level of sociality and the properties of the social relationship were varied (testing subjects alone = no sociality, in the presence of their partner = high sociality/intimate other, and in the presence of the experimenter = high sociality/formal other).

### 2.1. Subjects

In study 1, 63 University of Bamberg (Germany) students were recruited via advertisements posted in the university buildings. Participants were randomly assigned to be tested either in a situation of moderate sociality (nonvisual interaction; ♀ = 15 and ♂ = 17; mean age = 23.3 ± 4.4 years) or of high sociality (visual interaction was possible; ♀ = 17 and ♂ = 14; mean age = 22.4 ± 4.2 years). In study 2, couples who had been in a relationship for more than 6 months were recruited via advertisements in the local newspaper. All of the 100 participants (50 couples; mean age = 34.7 ± 10.8 years) were tested in three different social situations: alone (nonsocial), in the presence of the partner (high sociality/intimate other), and in the presence of the experimenter (high sociality/formal other) (see Table 1). 

Exclusion criteria in both studies were the current experience of acute or chronic pain and any kind of major diseases. None of the participants had taken analgesics, psychotropic medication, or alcohol the day before testing. All participants provided an informed consent and received either course credits (study 1) or monetary compensation (study 2) for their participation. The studies were approved by the ethics committee of the University of Bamberg.

### 2.2. Stimulation

In both studies, pain was induced by the use of a Peltier-based, computerized thermal stimulator (Medoc TSA-2001; Medoc Ltd, Ramat Yishai, Israel). The contact probe (3 × 3 cm^2^) was attached to the left lower leg. To ensure that the temperature intensities were perceived as painful but not too painful in all subjects (in order to prevent floor as well as ceiling effects), temperature intensities were tailored to the individual's pain threshold. Thus, heat* pain thresholds* were determined first using the method of adjustment. Subjects were asked to adjust a temperature starting from 38°C, using heating and cooling buttons, until they obtained a level which was barely painful. A constant press of the buttons produced a heating or cooling rate of 0.5°C/s. Following a familiarization trial, there were 5 trials and the average of these trials was taken as the pain threshold. Following the assessment of pain thresholds, phasic heat stimuli 5 s (plateau; rate of change: 4°C/s; baseline temperature: 38°C; interstimulus intervals of 20–25 s) were applied to the lower leg. Two different stimulus intensities were applied, namely, painful (+3°C above the pain threshold) as well as nonpainful (−3°C below the pain threshold). Applying nonpainful intensities allows the determination of the degree to which smiling during thermal stimulation was specific for painful experiences. In each experimental block of studies 1 and 2, participants always received 10 painful and 10 non-painful stimuli in a random order.

### 2.3. Dependent Variables

#### 2.3.1. Self-Report Ratings

In both studies, participants were asked to rate the intensity of their pain (see Table 1) on visual analogue scales (VASs). The VAS used in study 1 was labelled with verbal anchors from “no pain” (0) to “extremely strong pain” (100). In order to also assess more differentially the non-painful intensities, the VAS used in study 2was labeled with the verbal anchor “faintly painful” in the centre so that all non-painful sensations were rated below 50 and all painful ones at 50 or above (subjects were instructed that the lower end meant no felt change in the temperature and the upper end would indicate extremely strong pain). Subjects provided ratings after each stimulus. 

#### 2.3.2. Facial Measurement

**Figure 1:**
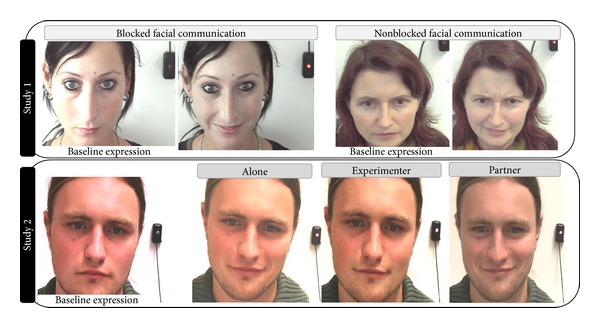
Examples of smiles (AU 12) that occurred during painful stimulation in study 1 (upper row: examples are given for the blocked and nonblocked facial communication groups) and during study 2 (lower row: examples are given for the three social situations participants were tested in).

In both studies, the faces of the participants were videotaped throughout the pain induction procedures. The camera was located on top of the computer screen, and subjects were told that besides their self-report of pain, we were also assessing their behavioral responses to pain. A LED was visible on the camera but not to the participant, was illuminated concurrent with the phasic thermal stimuli to mark the onset of stimulation (see also Figure 1). Smiling was coded from the video recordings using the Facial Action Coding System [6], which is based on anatomical analysis of facial movements and distinguishes 44 different Action Units (AUs) produced by single muscles or combinations of muscles. We focused only on the occurrence of Action Unit 12 (AU 12; lip corner raise), the product of zygomaticus major activity. AU 12 is the principal movement recognized by observers as indicating a smile. Ekman et al. [12] showed that the intensity and duration of this action were associated with self-reports of happiness and predicted which of the two emotionally pleasant films people reported making them more happy. Harris and Alvarado [13] showed that AU 12 combined with AU 6 (contraction of orbicularis oculi) in the form of a “Duchenne smile” [14] occurred more frequently while watching comedy sketches and being tickled than during cold pressor pain. Since AU 6 is also a main component of pain expressions making it difficult to decide whether the orbicularis oculi activation is part of the pain or part of the smile expression, we decided to focus exclusively on AU 12 in the present studies.

In both studies, the frequencies and intensities of AU 12 during all painful and non-painful stimuli were coded. Intensity is coded on a 5-point scale, ranging from “trace” to strong. To segment videos and to enter the FACS codes, we used the Observer Video-Pro (Noldus Information Technology). Time segments of 5 s (plateau duration of each stimulus) were selected for scoring. We scored AU 12 whenever it occurred (regardless of whether other AUs occurred simultaneously or not). The data were FACS coded by 5 coders. In order to calculate interrater reliability, a certified FACS Coder (MK) coded 5% of video segments coded by each of the 5 coders. Interrater reliability, as calculated using the Ekman-Friesen formula ([6] number of AU 12 agreed upon × 2 divided by the overall occurrences of AU 12 coded), ranged from 0.89 to 0.94, which compares favourably with other researches in the FACS literature. For further analyses, product scores for AU 12 (intensity × frequency) were computed. This was done separately for painful and non-painful stimulation. As a last step, the product scores for AU 12 were square-root-transformed given their skewed distribution across subjects. Examples of AU 12 can also be seen in Figure 1.

### 2.4. Procedures

#### 2.4.1. Study 1 (Slight Differences in Sociality)

In study 1, the degree of smiling was compared between two levels of sociality: high versus moderate. The experimenter was always in the room with the participant. The differences between the conditions were that the experimenter either faced the participant throughout the experimental procedure (visual interaction was possible) or the experimenter sat behind a computer screen so that no eye-contact was possible throughout the experimental procedure (nonvisual interaction). Prior to reporting to the laboratory, participants were assigned at random to one of the two conditions. Participants were informed that they would receive 20 thermal stimuli of different intensities and that after each stimulus they should provide a VAS rating (the VAS-scale appeared on the screen after each stimulus).

#### 2.4.2. Study 2 (No Sociality versus High Level of Sociality and Intimate versus Formal Other)

In study 2, we additionally varied the properties of the social relationship. Besides testing participants while they were alone in the room (no sociality), we varied two other types of social situations by testing participants in the presence of the experimenter (formal other) and in the presence of their partner (intimate other). In both social situations, the partner/experimenter was seated such that he/she was facing the participant, thus representing situations with high sociality. Given that we only included couples that had been in a partnership for more than six months, we feel justified to label the partner as an intimate other. To increase statistical power, we used a within-subject design: each participant was tested in each of the three situations. The order of social situations was balanced across participants. (Given that we investigated both partners, subjects participated in the “intimate other” situation twice: once while experiencing pain and once while observing the partner. To exclude the possibility that participants' smiling during the “intimate other” situation was not affected by having observed the partner before, we correlated the occurrence of smiling between partners and found no significant correlations.) Participants were told that we were interested in how pain perception changes across time and across social situations. In the nonsocial condition, subjects were alone in the experimental room but they knew that their faces were being videotaped. Participants were instructed not to talk during the stimulation. In all three conditions, participants were informed that they would receive 20 thermal stimuli of different intensities and that after each stimulus they should provide a VAS rating (VAS scale appeared on the screen after each stimulus). To avoid sensitization, the site of stimulation was changed after each social condition.

### 2.5. Statistical Analysis

In study 1, the impact of slight variations of the level of sociality on subjective ratings and on the occurrence of smiling was investigated using the analyses of variance with one within-subject factor (stimulus intensity_non-painfulheat,painfulheat_) and one between-subject factor (level of sociality_nonvisualcommunication,visualcommunication_). 

In study 2, the impact of different levels of sociality and of different properties of the social relationship on subjective ratings and on the occurrence of smiling was investigated using analyses of variance with 2 within-subject factors (stimulus intensity_non-painfulheat,painfulheat_), type of social situation_alone,withtheexperimenter,withthepartner_). Where significant effects were found, *t*-tests were calculated for single comparisons. Findings were considered to be statistically significant at *α* < 0.05.

## 3. Results

### 3.1. Study 1 (Slight Variations in the Level of Sociality)

The pain threshold did not differ between participants being observed by the experimenter (mean: 45.9°C; SD: 1.5) and those not being observed (mean: 45.9°C; SD: 1.1) (*P* > 0.05). 

#### 3.1.1. Self-Report

We found a significant main effect for stimulus intensities on VAS ratings (*F*(1,61) = 1034.08; *P* < 0.001). As can be seen in Table 2, VAS ratings for the painful stimuli were significantly higher than for the nonpainful stimuli. The social situation (high sociality versus moderate sociality) yielded no significant effect (*F*(1,61) = 0.39; *P* = 0.536) and did not interact with stimulus intensity (*F*(1,61) = 0.68; *P* = 0.412).

#### 3.1.2. Smiling

**Figure 2:**
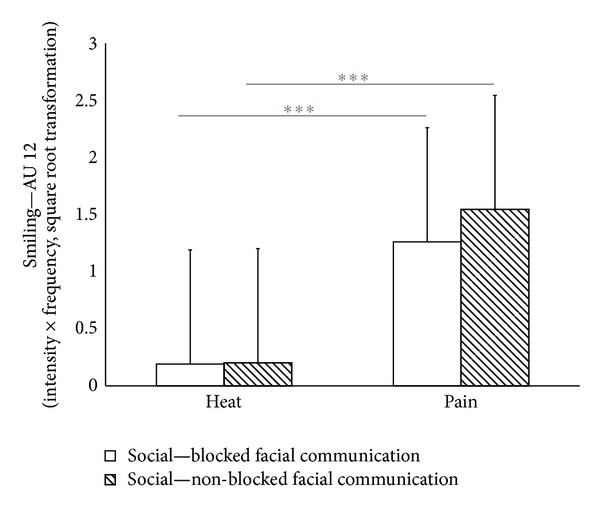
Degree of smiling (AU 12) (mean values (+SD) while undergoing non-painful and painful heat stimulation (study 1)). Values are given separately for the group of subjects being seated in sight of the experimenter (visual interaction) and for the group unobserved by the experimenter (nonvisual interaction). *(Moderate *(*d* > 0.5)* and strong *(*d* > 0.8)* effect sizes between stimulus intensities are marked in bold.) *

As can be seen in Figure 2, AU 12 significantly increased during painful heat stimulation compared to the non-painful stimulation (*F*(1,61) = 45.83; *P* < 0.001). However, the degree of smiling did not vary between participants being observed by the experimenter and those not being observed (*F*(1,61) = 0.45; *P* = 0.508) (see also Figure 1 for examples of smiling occurring during painful stimulation in both groups). Thus, slight variations of the level of sociality (by restricting the visual interaction) did not lead to differences in smiling during pain, nor did the social situation interact with the impact of stimulus intensity (*F*(1,61) = 0.58; *P* = 0.449). Overall, smiles occurred in 21% of all pain trials across all subjects.

### 3.2. Study 2 (No Sociality versus High Level of Sociality and Intimate versus Formal Other)

The mean pain threshold in study 2 was 46.1°C (SD: 1.2) and did not differ from the thresholds of study 1 (*P* > 0.05). Thus, stimulus intensities were comparable between the two studies. 

#### 3.2.1. Self-Report

We found a significant main effect for stimulus intensity (*F*(1,99) = 1235.64; *P* < 0.001). As can be seen in Table 2, VAS ratings for the painful stimuli were significantly higher than for the non-painful stimuli. The social situation (alone versus intimate other versus formal other) yielded no significant main effect (*F*(2,198) = 1.48; *P* = 0.231). However, both factors (stimulus intensity and social situation) significantly interacted with each other (*F*(2,198) = 5.10; *P* = 0.007). Post hoc *t*-tests revealed that this interaction was due to the ratings for the non-painful stimuli being significantly higher when tested with the partner compared to the other two situations (*P* < 0.05; see Table 2). 

#### 3.2.2. Smiling

**Figure 3:**
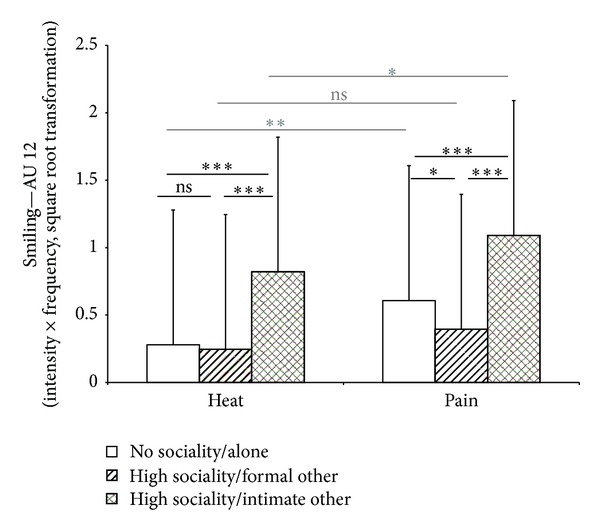
Degree of smiling (AU 12) (mean values (+SD) while undergoing non-painful and painful heat stimulation (study 2)). Values are given separately for the three situations (being alone (no sociality), being with the partner (high sociality/intimate other), and being with the experimenter (high sociality/formal other)). *(Moderate *(*d* > 0.5)* and strong *(*d* > 0.8)* effect sizes are marked in bold. Effect sizes for the differences in stimulus intensities are given in gray, whereas differences between situations are given in black.) *

As can be seen in Figure 3, AU 12 significantly increased during painful heat stimulation compared to the non-painful stimulation (*F*(1,99) = 27.26; *P* < 0.001). Moreover, the degree of smiling also varied depending on the social situation the participant was tested in (*F*(2,198) = 16.09; *P* < 0.001). As can be seen in Figure 3, smiling occurred significantly more often when painful stimuli were applied in the presence of the partner, whereas smiles were shown less frequently when subjects were tested in the presence of an experimenter (see also Figure 1 (lower row) where an example is given). We also found a significant interaction between stimulus intensity and social situation (*F*(2,162) = 9.97; *P* < 0.001). As post hoc *t*-tests revealed, the increase in AU 12 from non-painful to painful intensities was only significant when participants were tested alone or in the presence of the partner (*P* < 0.05; see also Figure 3), whereas smiling occurred to a similar degree during non-painful and painful stimulation when participants were in the presence of the experimenter (*P* > 0.05). Overall, smiles occurred in 19% of all pain trials across all subjects and across all social situations.

## 4. Discussion

The aim of the present studies was to investigate whether the seemingly incongruent facial expression “smile” occurring during the experience of pain belongs to the group of *affiliate smiles* [8] that serve the purpose of communicating social motives (e.g., initiating social interactions or strengthening and maintaining social bonds). To evaluate the social motives account of smiling during pain, two studies were conducted in which the level of sociality was systematically varied and different properties of the social relationship were considered. 

### 4.1. Subtle Changes in the Level of Sociality

In a first approach, we were interested in investigating whether subtle manipulations of the level of sociality would have an effect on the degree of smiling during pain. All participants experienced stimulation in the presence of an experimenter; however, in one condition, visual interaction between the two was possible (experimenter faced the participant), while in the other, visual interaction was blocked by a computer screen. These more subtle variations in the level of sociality did not affect the degree of smiling. Regardless of whether visual interaction was blocked or was not blocked, the magnitude of smiling during pain was similar. If social motives underlie smiling during pain (as has been assumed for the group of *affiliate smiles* [8]), one would expect its occurrence to change depending on whether visual communication is available or, in other words, whether the smile can indeed be used for social interaction. However, it might be that simply knowing that somebody else is in the room, and therefore knowing that visual communication is potentially possible, have a very similar effect to being directly observed. Interestingly, Fridlund and colleagues [15, 16] pointed out that the degree of smiling (in response to positive experiences) increases even when individuals are only imagining being in the presence of someone they know compared to being alone. Therefore, social motives might already evoke smiling during the experience of pain when the social other is just being imagined or just present in the same room, regardless of whether direct visual interaction is taking place or not. Another explanation for finding the degree of smiling to be unaffected by subtle changes in the level of sociality might be that the direct eye gaze of the experimenter was experienced as aversive (as has been shown, for example, in social phobic individuals [17, 18]), and thus, the increased sociality due to the eye-contact with the experimenter might have been counteracted by an increased aversiveness of this social setting.

### 4.2. Substantial Changes in the Level of Sociality (No Sociality versus High Sociality) and Properties of the Social Relationship

In the second study, we decided to investigate whether smiling during pain is affected by more substantial changes in the level of sociality as well as by the properties of the social relationship between interactants. To do this, participants experienced three separate conditions in a repeated measures design: a nonsocial condition in which they experienced heat and pain while being alone, a condition in which they were aware of an observing experimenter (high sociality/formal other), and a condition in which they experienced stimulation in the presence of a person with whom they were familiar or intimate (high sociality/intimate other). We found that the degree of smiling during pain varied as a function of stimulus intensity, level of sociality, and properties of the social relationship. 

#### 4.2.1. Level of Sociality and Properties of the Social Relationship

Our data clearly show that a high level of sociality does not lead to increased smiling during pain per se. Instead, smiling was even diminished if subjects were in the presence of the experimenter, whereas smiling occurred quite frequently when participants were being observed by their partner. This finding is consistent with the assumption that social motives should differ depending on whether one interacts with a friend/partner or a stranger [19]. Moreover, this finding would also be in line with the assumption that smiling during pain belongs to the group of *affiliative smiles* that serve the motives of initiating social interactions or strengthening and maintaining social bonds [8]. Given that pain is an unpleasant experience [10], social bonding might be especially important in this situation. Individuals might smile during the experience of moderate pain intensities in the presence of their partner in order to elicit positive emotions in their partner, thereby increasing positive social interactions, empathy, and potential support. Indeed, it has been shown that the display of smiles during negative affect (sadness) was associated with more positive emotions and less frustration in observers as well as with the self-reports of better relationships [20]. The relationship between participant and the experimenter, on the other hand, was of a completely different nature. It was solely professional in this study, and thus, the participants might not have felt the need to display smiles. Moreover, it seems likely that the existence of salient role and/or status differences between experimenter and participant defined a more formal social relationship, more likely to promote self-monitoring and suppression on the part of the participant [21]. The findings suggest that being in the presence of the experimenter inhibits the degree of smiling, whereas the presence of an intimate partner tends to “release” smiling during painful stimulation. Accordingly, the level of sociality alone does not seem to be the crucial factor, but instead the relationship between sending and receiving persons seems to determine whether smiles are shown in response to pain.

Surprisingly, smiling was also displayed when participants were alone in the room, though to a lesser degree than in the partner situation. This finding is less consistent with the assumption that smiling during pain belongs solely to the group of *affiliative smiles*. However, as stated in the introduction, it is possible that smiling during pain—besides belonging to the group of *affiliative smiles*—is also part of an opponent intrapersonal process, namely, a self-regulatory process that counterbalances the negative affective state [4, 9, 22, 23], given that smiling has been shown to reduce perceived pain intensity making individuals more pain tolerant [24]. Thus, smiling during pain might not only have social but also nonsocial determinants. However, this interpretation is only speculative, since we did not assess whether smiling leads to an improved mood or less unpleasantness during the painful experiences. Furthermore, it is possible that subjects did not really feel completely alone, given that they knew that their face was being videotaped.

#### 4.2.2. Level of Intensity/Specificity of the Smile of Pain

In both studies, we also applied non-painful heat intensities to investigate whether smiles indeed occur more often during painful stimulation and to exclude the possibility that smiles during pain are only an experimental artifact. In both studies, smiling did differ according to the nature of the stimulus delivered with participants smiling more when the thermal stimulation was painful than when it was not. Thus, there is indeed a link between the painful quality/intensity of the stimulation and the smiling behavior. Consequently, it is not the experimental context by itself that elicits smiling, but indeed something about the painful intensity of the thermal stimuli. 

### 4.3. Limitation

These studies were a first attempt to investigate why smiling might occur in the context of pain by focusing on potential social motives. However, we did not directly assess social motives but tried to manipulate them indirectly by changing the social context. Future research should try to assess more directly the social motives underlying the occurrence of smiling during pain (e.g., by including self-report measurements). Furthermore, although our findings suggest that the smile during pain belongs to the group of *affiliative smiles,* we cannot refute that the smile occurring during pain might instead belong to a whole new category of smiles. As mentioned before, the occurrence of smiling during pain (similar to the occurrence of smiling during disgust and sadness [23]) might have the discrete function to counterbalance the negative affective state, and thus might be classified as “*opponent processes smiles*.”

## 5. Conclusion

We found that the occurrence of smiling while experiencing non-painful and painful heat stimuli differed depending on the intensity of stimulation, the level of sociality, and the properties of the relationship between interactants. Most robust findings were found for the level of stimulus intensity, with smiling occurring much more frequently, intensely, and enduringly during painful compared to non-painful stimulation. Moreover, the properties of the relationship had a considerable impact, with smiling being reduced in the presence of a formal other, whereas the presence of the partner significantly increased the smiling behavior. Like smiles during other types of nonenjoyment states, the smile of pain might be less of a sign of the underlying affect than a reflection of social motives. Future studies have to investigate which impact the occurrence of smiling during pain has on the observer. Does it really strengthen social bonds or is the observer rather irritated by this seemingly pain-incongruent facial expression? Given the importance of the facial communication of pain in social interactions (elicitation of solicitous behaviors and empathic responses in the observers) and for pain diagnostic and pain treatment [25], it would be very disadvantageous for the person in pain if smiling during pain elicits a feeling of irritation in the observer.

## Figures and Tables

**Table 1:** Overview of the experimental procedures and dependent variables of the 2 studies.

Procedures and variables	Study 1 Degree of sociality (*N* = 63)	Study 2 Degree of sociality and properties of the social relationship (*N* = 100)
Design	Between-subject design	Within-subject design
Social manipulation	(i) Moderate sociality (nonvisual interaction) (ii) High sociality (visual interaction possible)	(i) No sociality (alone) (ii) High sociality—formal (with the experimenter) (iii) High sociality—intimate (with the partner)
Pain induction	Phasic heat stimuli (painful and nonpainful)	Phasic heat stimuli (painful and nonpainful)
Assessment of smiling	AU 12 (frequency and intensity being coded)	AU 12 (frequency and intensity being coded)
Self-report rating	VAS (0–100)	VAS (0–100; with 50 being labelled as slightly painful)

**Table 2:** Self-report ratings (mean values ± SD) in the two studies.

		**No sociality**	**Moderate sociality**	**High sociality**	
			with the experimenter—nonvisual interaction	with the experimenter—visual interaction possible	
Study 1					
Ratings (VAS)	Nonpainful	—	1.6 (±3.1)	2.2 (±5.1)	
	Painful	—	62.8 (±14.9)	65.9 (±17.1)	

		Alone		With the experimenter	With the partner

Study 2					
Ratings (VAS)	Nonpainful	14.2 (±12.9)	—	16.3 (±14.2)	18.8 (±14.2)
	Painful	81.4 (±12.1)	—	80.2 (±12.8)	80.2 (±13.0)
